# Behavioral evidence for global consciousness transcending national parochialism

**DOI:** 10.1038/s41598-023-47333-z

**Published:** 2023-12-04

**Authors:** James H. Liu, Sarah Y. Choi, I-Ching Lee, Angela K.-y. Leung, Michelle Lee, Mei-Hua Lin, Darrin Hodgetts, Sylvia Xiaohua Chen

**Affiliations:** 1 Massey University, Albany, New Zealand; 2 National Taiwan University, Taipei, Taiwan; 3 Singapore Management University, Singapore, Singapore; 4 Sunway University, Selangor, Malaysia; 5 Hong Kong Polytechnic University, Hong Kong, China

**Keywords:** Evolution, Psychology, Environmental social sciences

## Abstract

While national parochialism is commonplace, individual differences explain more variance in it than cross-national differences. Global consciousness (GC), a multi-dimensional concept that includes identification with all humanity, cosmopolitan orientation, and global orientation, transcends national parochialism. Across six societies (N = 11,163), most notably the USA and China, individuals high in GC were more generous allocating funds to the other in a dictator game, cooperated more in a one-shot prisoner’s dilemma, and differentiated less between the ingroup and outgroup on these actions. They gave more to the world and kept less for the self in a multi-level public goods dilemma. GC profiles showed 80% test–retest stability over 8 months. Implications of GC for cultural evolution in the face of trans-border problems are discussed.

## Introduction

Today, the political economy of the world is organized in such a way that state-based sovereignty is foregrounded within global structures that emphasize national parochialism- or ingroup favoritism at the level of the state^1^. Concurrently, cooperation in trade across national borders is taking place at a scale unprecedented in history^2^. While there has been some experimental research on cooperation across national boundaries^3,4^, most of the literature is based on self-report measures, and/or data from only Western countries^5^. However, new research using the prisoner’s dilemma game showed that national in-group favoring resource allocations by individuals was almost “ubiquitous” across 42 countries^6^. That is, it was significant and consistent in magnitude across 39 of 42 countries ranging from Sweden to Nigeria. The extent of variation in national parochialism at the country-level could not be predicted by important scientific theories, including pathogen stress, prevalence of modern world religions, rule of law, and governmental effectiveness^6^. From a global perspective, this suggests that the state is a collective capable of carrying in-group favoritism, regardless of whether this state is a superpower or politically fragile^7,8^.

Furthermore, while it is well-known that national parochialism stands as an obstacle to managing cross-border issues from climate change to pandemic prevention, it is not well-understood what offers a way forward for dealing with what appears to be a human “tribal instinct”^9^. Tribal instincts to favor the national ingroup appear commonplace, but it is *not* true that the extent of national parochialism in a given country is homogeneous. Rather, individual and group differences within countries explain more of the variance in national parochialism than differences between countries^6^. An analysis of individual differences using the same 42 country dataset cited previously found that conservatives showed more parochialism than liberals, and that this effect was more pronounced in countries with higher quality of institutions (including having better rule of law and higher levels of socio-economic development)^10^. However, the limitation on this finding is that liberalism-conservatism is a measure of local differences in political attitudes/ideology, and hence cannot offer a solution to the global challenge to go beyond internal divisions and beyond national parochialism^39^. Rather, this feeds into other forms of parochialism and in-group favoritism that divide people into groups *within* countries^11,12^.

### The rise of global consciousness (GC)

Fortunately, advances have been made recently on one form of individual differences that has both profound and logical implications for transcending national parochialism. This literature situates moral development^42^ into a group-based context where moral concerns about intergroup relations and environmental sustainability are emphasized. The literature is most famously referred to as cosmopolitanism^13–15^ (by the ancient Greeks, and enlightenment philosopher Immanuel Kant), and more recently as identification with all humanity^16,17^ (or global citizenship)^18^. Different researchers have developed different measures, each sophisticated, and each anchored to different literatures. The best-known conceptual measure is rooted to the social identity literature in psychology and focuses on sense of inclusion of self as part of all humanity and concern for all humanity [IWAH^16,17,19,45,46^]. A recent review reported that IWAH is associated with prosocial values, charitable behavior and behavioral intentions, lower prejudice, and pro-environmental attitudes and intentions^17^. However, different global identities (such as global citizenship and human identities) have been shown to represent different prototypical meanings, and to have different empirical correlates^44^. Beyond this, the literature on cosmopolitanism, spread out across the humanities and social sciences, is even more extensive in the breadth of its implications. An analysis of this literature revealed that cultural openness, global pro-sociality, and respect for cultural diversity constitute the central dimensions of cosmopolitanism at the individual level, and all three of these had impacts on prosocial behavior, especially environmental conservation^15^. Finally, a critical context for the expression of GC is social interactions across cultures; in this area (rooted in the literature on acculturation and cross-cultural psychology), a proactive orientation towards multicultural learning, and lower concern about ethnic in-group protection are central^20^.

Conceptually, what pulls all these meanings together is a philosophical core, drawing from the literature surrounding Emmanuel Kant’s ideas about what it means to be a citizen of the world^13,14^. Global consciousness (GC)^21^ is defined as “a knowledge of both the interconnectedness and difference of humankind, and a will to take moral actions in a reflexive manner on its behalf” (p. 310). Global consciousness encapsulates the understanding and appreciation of cultural diversity, human connectedness, and proactive measures towards inter-cultural interactions. GC has meanings that encompass a family of measures sharing some common conceptual core, but also with disparate elements that are more or less salient across different situations, as represented by different literatures. GC is therefore conceptualized as a multidimensional construct, composed of lower order factors^48^. It carries a lifelong developmental trajectory that may be related to adaptive intelligence^28^ and the development of a moral identity^43^.

Previous research has shown that the various dimensions of GC are modestly positively correlated across 35 cultures^40^, and that the operationalization of GC has been well validated in longitudinal research. A parallel study drawing from the same dataset, but using a different sample and focusing on demographics and life events has recently been accepted for publication^41^. Operationally, the current research incorporates all three measures using latent profile analysis^22^. Latent profile analysis (LPA) provides a highly reliable, temporally stable, and person-centered measure of GC. It is appropriate for measuring GC because while these three measures do have a common core, it is unclear if the concept itself is a second-order latent variable, or whether it is more complex (i.e. situationally dynamic, or a higher order variable). Preregistration for this study focused on the behavioral impacts of GC as a person-level variable using LPA, rather than using a latent variable strategy. Combining the different dimensions of GC into latent profiles that differentiate one person from one another is the best way to capture the behavioral impacts of GC. However, the dimensions of GC analyzed separately for their impacts on outcome variables are also provided in supplementary materials S1.

Measurement of GC as a construct is new, and research using different operationalizations have demonstrated its external validity mainly on self-reported attitudes, values, behaviors, and intentions. Given the potential of social desirability bias, it is important to test the impact of GC on behavioral dependent measures and confirm that these impacts hold over time.

Game theoretical approaches have long been used as experimentally controlled measures of cooperative behavior^23^ that can gauge the impact of global consciousness in social exchange. Three games stand out because they have clear analogies to important real-world phenomena. These are (a) the dictator game^24^, which is a useful analogy for charitable giving or altruism^25^, (b) the prisoner’s dilemma game, which has long been used to model cooperative actions involving risk such as economic exchange^26^, and (c) the multi-level public goods dilemma, purpose-built to examine global cooperation and environmental sustainability^3^. Charitable giving, economic exchange, and contributions to public goods are three central behavioral domains where the impact of global consciousness should be visible^21^.

We report two longitudinal online experiments of two waves, each testing one behavioral consequence of global consciousness. In Experiment one, wave 1 (from July 2 to July 16, 2020) involved 6138 participants, with 1449 participants retained in wave 2 (administered from October 5 to November 8, 2020). In Experiment two, wave 1 (from March 28 to May 7, 2021) involved 4925 participants, with 1459 retained in wave 2 (from July 5 to August 8, 2021). The total sample across experiments included 11,063 participants from China, Hong Kong, Malaysia, Singapore, Taiwan, and the United States, with data collection stratified for gender, age, and socio-economic status. These locations were chosen because in addition to the pre-registerered behavioral research described here, another purpose of the overall research enterprise (not described here) was to explore the impact of non-Western constructs on the phenomena being investigated^27^.

In choosing our samples, we began with the idea to extend the literature on global consciousness beyond the North American and European societies where most work on it has originated^17^. Because published literature is more robust in documenting psychological and behavioral tendencies of people in Western societies who are culturally similar to the USA^38^, we extended this investigation to also encompass other Asian societies more culturally similar to China. We provide a direct comparison of the USA and China, and also aggregate results of these two societies together with Hong Kong, Malaysia, Singapore, Taiwan for an omnibus test of preregistered hypotheses.

Hypotheses on the relationship between global consciousness (GC) and various forms of national parochialism were pre-registered, and the original data can be downloaded on https://osf.io/6yebu/).

First, people high in GC were hypothesized to give more to charity and to have a smaller difference between giving to a national ingroup versus to an outgroup. Second, they were hypothesized to give more to the ‘other’ in the prisoner’s dilemma, and to have a smaller difference between giving to a national ingroup versus outgroup member. Third, they were hypothesized to allocate more resources to the world compared to self, and to the local group compared to the self in a multi-level public goods dilemma. We changed the pre-registered 3 wave design to 2 Experiments of 2 waves each to compensate for a higher than anticipated attrition rate. The longitudinal design enabled us to avoid possible priming effects of GC, by measuring dependent variables months later in Wave 2 (this included all dependent measures except charitable giving). Although we measured GC in both waves, we used GC at Time 1 as a predictor for behavioral outcomes at Time 2 in our longitudinal results for a more stringent predictive test across time.

In charitable giving (Experiment 1, wave 1), participants took part in a within-subjects experiment with two counterbalanced trials, having the reward structure of a dictator game. In one condition they were paired with a real ingroup environmental charity in their own society/country. In the other (outgroup condition) they were paired with the World Wildlife Fund in New Zealand. In each trial they were asked what percentage of the allocated amount they would keep for themselves. In all the economic games reported in this research, participants were allocated approximately 1 USD per trial, with small adjustments according to purchasing power parity across countries. The remainder was given to the charity.

In the prisoner’s dilemma game (PDG; Experiment 1, wave 2 and Experiment 2, wave 1), participants took part in a two-trial within-subjects experiment, with two counterbalanced conditions. In one condition, they were told that they were paired with an ingroup member. In the outgroup condition, they were told they were paired with someone from one of the other countries/societies, without specifying which one. In each trial they were asked “What percentage do you keep for yourself, and how much do you want to allocate to your partner, who is from [ingroup/several outgroups]? Your partner also has the same decision about how much to allocate to you. The money you allocate to one another will be doubled.” The amount they received from the partner was double the average allocation given to ingroup members (in the ingroup trial) and double that allocated to outgroup members (in the outgroup trial). They received this amount in addition to the amount they kept for themselves, maintaining the mathematical structure of the PDG.

Finally, in the multi-level public goods dilemma (MLPG; Experiment 2, wave 2), participants were given two trials. They were told they were in a group with 11 other persons in the experiment, from the six locations described. It was explained that everyone in the group was allocated the same amount, and faced with the same decision, which is as follows:

“Please decide how many points to keep for yourself, how many to contribute to other people from your home region (LOCAL ACCOUNT), and how much to give to other people from around the world (WORLD ACCOUNT). The total points in the 3 boxes must equal 10.”

It was explained that points allocated to the local account were to be doubled, and divided equally amongst the participants in that country/society. Points allocated to the world account were to be tripled, and divided equally among all participants. This is a classic public goods dilemma^3^ where everyone prospers if they give more to the collective, but simultaneously each individual is motivated to allocate more to themselves or their local ingroup. Participants were actually allocated exactly the amounts warranted by their decisions and those of the other participants in the game.

Because of the complexity of the games, participants were always given a trial round that did not count, where they were given feedback and shown how to calculate their hypothetical outcomes given example choices prior to them making the behavioral decisions involving actual rewards.

**Figure 1:**
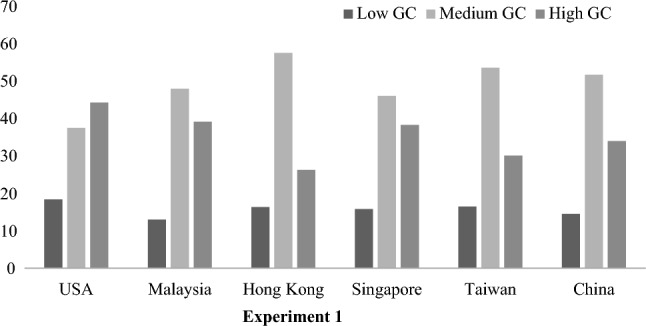
Overall distribution (%) of global consciousness (GC) profiles across societies.

A series of one-way analyses of variance were performed to examine the effects of global consciousness on our dependent measures of interest. Alternative analyses were performed using Cosmopolitan Orientation, Global Orientations and Identification With All Humanity scales as separate predictors for the behavioral outcomes (see supplementary materials S1, Tables S12 and S13). This showed that only for the Multi-level Public Goods Dilemma were all three scales significant as separate predictors. The latent profiles were much more robust predicting all behavioral outcomes than the separate predictors. This variable consists of 3 profiles, high, medium, and low, derived via latent profile analysis^22^ that is robust across cultures, and had 80% stability over 8 months (see Fig. 1, and supplementary materials S1). Such test–retest stability is on par with that of established measures of personality^37^, though GC is conceptualized as a feature of character, that can be cultivated over the lifespan^21^, rather than simply genetically inherited. All main effects reported here remained the same when controlling for demographic covariates (age, gender, education, and subjective SES) unless commented otherwise.

## Experiment 1

### Global consciousness (T1) predicting charitable giving in dictator game (T1)

#### Ingroup charity

There was a small significant main effect of global consciousness (GC) at Time 1 on amount donated to an ingroup charity at Time 1 (*F*^2^ = 22.27, *p* < 0.001, η^2^ = 0.007) in the charitable giving task. The mean amount donated to ingroup charity was significantly *higher* for those with high (M = 0.344, SD = 0.281) compared to low (*M* = 0.274, *SD* = 0.286) and medium (M = 0.307, SD = 0.281) GC (*p*s < 0.001). The mean amount donated to ingroup charity was also significantly higher for those with medium compared to low GC (*p* = 0.008).

#### Outgroup charity

**Figure 2:**
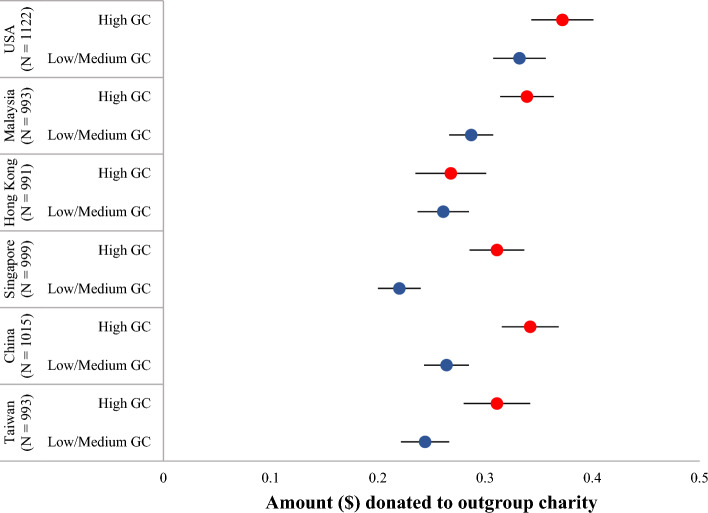
Mean amount allocated to outgroup charity by high compared to low and medium Global Consciousness (GC) profiles across countries/societies. Societies are organized by high to low scores in individualism; error bars represent 95% confidence intervals; low and medium GC profiles are aggregated for display purposes.

There was also a small significant main effect of GC (Time 1) on amount donated to an outgroup charity (Time 1), (F(2, 6110) = 33.32, *p* < 0.001, η^2^ = 0.011). The mean amount donated to outgroup charity was significantly *higher* for those with high (M = 0.327, SD = 0.286) compared to low (M = 0.246, SD = 0.296) and medium (M = 0.275, SD = 0.283) GC (*p*s < 0.001). The mean amount donated to outgroup charity was also significantly higher for those with medium compared to low GC (*p* = 0.025). High GC participants donated the most to the outgroup charity across all countries/societies (see Fig. 2), and this was not significant only in Hong Kong, a Special Administrative Region of China, though the pattern of Hong Kong exhibited the same trend.

#### Parochial nationalism

**Figure 3:**
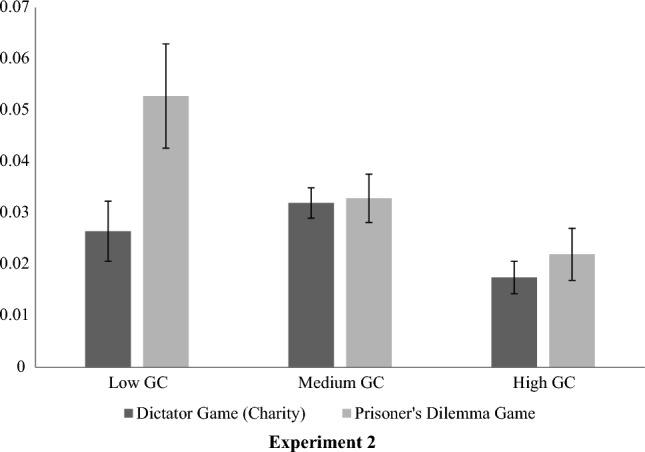
Parochial Nationalism (or ingroup favoritism) exhibited in the Dictator Game (T1) and Prisoner’s Dilemma Game (T2), across Global Consciousness profiles.

Finally, there was a small main effect, showing significant differences between GC profiles (Time 1) in amount donated to the ingroup compared to the outgroup charity (Time 1), (F(2, 2250.26) = 5.65, *p* = 0.004, *ω*^2^ = 0.002). For those analyses where the homogeneity of variance assumption was not met, the Welch test followed by Games-Howell’s post hoc comparisons (ω2) were provided instead of conventional effect sizes (η2). Ingroup favoritism scores were calculated by subtracting amount donated to outgroup charity from amount donated to ingroup charity. As seen in Fig. 3, parochial nationalism (i.e. ingroup favoritism) was significantly *lower* for those with high (M = 0.018, SD = 0.152) compared to medium (M = 0.032, SD = 0.16, *p* = 0.002) GC. Other comparisons were not significant (*p*s > 0.05).

### Global consciousness (T1) predicting prisoner’s dilemma game longitudinally (T2)

#### Ingroup partner

There were no significant differences between GC profiles at Time 1 on amount given to an ingroup partner in the Prisoner’s Dilemma Game (PDG) at Time 2, (F(2, 1446) = 0.38, *p* = 0.68).

#### Outgroup partner

There were also no significant differences between GC profiles (Time 1) in amount given to an outgroup partner in the PDG (Time 2), (F(2, 508.77) = 0.43, *p* = 0.65). These two results may be a function of the severely reduced sample size in wave 2.

#### Parochial nationalism

There was a small significant main effect of GC (Time 1) on amount given to an ingroup versus an outgroup partner in the PDG (Time 2), (F(2, 514.03) = 3.97, *p* = 0.02, *ω*^2^ = 0.004). As seen in Fig. 3, mean parochial nationalism (or ingroup favoritism) at Time 2 was significantly *lower* for those with high (M = 0.022, SD = 0.113) compared to low GC at Time 1 (M = 0.053, SD = 0.142, *p* = 0.019). Other comparisons did not reach significance (*p*s > 0.05).

## Experiment 2

### Global consciousness (T1) predicting prisoner’s dilemma game (T1)

#### Ingroup partner

In the second experiment, there was a small significant main effect of GC at Time 1 on amount given to an ingroup partner in the PDG at Time 1 (F(2, 2349.91) = 38.68, *p* < 0.001, *ω*^2^ = 0.015) using a more appropriately powered sample (attrition was higher than anticipated for wave 2 of Experiment 1). Mean amount given to an ingroup partner was significantly *higher* for those with high (M = 0.587, SD = 0.255) compared to low (M = 0.508, SD = 0.226) and medium (M = 0.53, SD = 0.225) GC (*p*s < 0.001). Mean amount given to an ingroup partner was also significantly higher for those with medium compared to low GC (*p* = 0.025).

#### Outgroup partner

**Figure 4:**
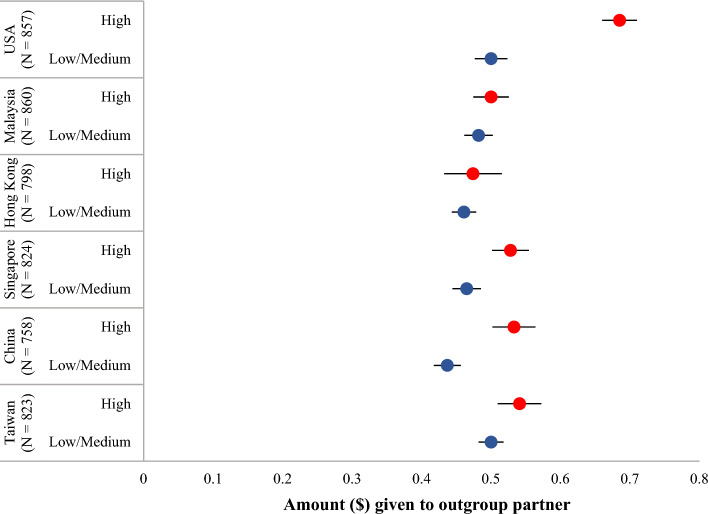
Mean amount allocated to outgroup partner by high compared to low and medium Global Consciousness (GC) profiles across countries/societies in the Prisoner’s Dilemma Game. Societies are organized by high to low scores in individualism; error bars represent 95% confidence intervals; low and medium GC profiles are aggregated for display purposes.

There was also a small significant main effect of GC (Time 1) on amount given to an outgroup partner in the PDG (Time 1), (F(2, 2327.58) = 70.08, *p* < 0.001, *ω*^2^ = 0.027). Mean amount given to an outgroup partner was significantly *higher* for those with high (M = 0.56, SD = 0.263) compared to low (M = 0.448, SD = 0.235) and medium (M = 0.483, SD = 0.228) GC (*p*s < 0.001). Mean amount given to an outgroup partner was also significantly higher for those with medium compared to low GC (*p* < 0.001). High GC participants allocated the largest amount to an outgroup partner across all countries/societies (see Fig. 4), and this was significant in 4 of the 6 societies while the patterns of the 6 societies exhibited the same trend.

#### Parochial nationalism

**Figure 5:**
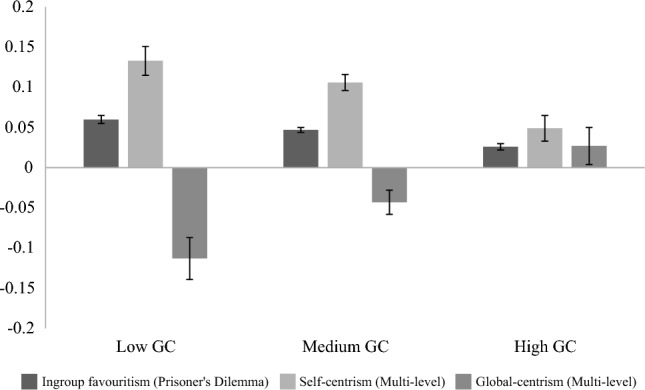
Mean ingroup favoritism (T1), self-centrism (T2) and global-centrism (T2) displayed across Global Consciousness profiles (T1) in Multi-level Public Goods Dilemma.

There were also significant differences by GC (Time 1) in levels of parochial nationalism displayed in the PDG (Time 1), indicated by a small main effect (F(2, 2324.08) = 16.41, *p* < 0.001, *ω*^2^ = 0.006). As seen in Fig. 5, mean ingroup favoritism was significantly *lower* for those with high (M = 0.026, SD = 0.147) compared to low (M = 0.06, SD = 0.153) and medium (M = 0.047, SD = 0.145) GC (*p*s < 0.001). There was no significant difference between low and medium GC profiles (*p* = 0.09).

### Global consciousness (T1) predicting multi-level public goods (MLPG) dilemma longitudinally (T2)

#### Myself

There was a small significant main effect of GC at Time 1 on amount reserved for self in the MLPG at Time 2, (F(2, 625.66) = 9.69, *p* < 0.001, ω^2^ = 0.012). The mean amount reserved for self, was significantly *lower* for those with high (M = 0.341, SD = 0.19) compared to low (M = 0.416, SD = 0.256, *p* < 0.001) and medium GC (M = 0.383, SD = 0.22, *p* = 0.003). There was no significant difference between low and medium GC profiles at Time 1 in amount reserved for self at Time 2 (*p* = 0.14).

#### Local group

There were no significant differences between GC profiles (Time 1) in amount allocated to the local group in the MLPG (Time 2), (F(2, 613.6) = 1.42, *p* = 0.24).

#### World

There was a small significant main effect of GC (Time 1) on amount allocated to the world in the MLPG (Time 2), (F(2, 1456) = 6.19, *p* = 0.002, η^2^ = 0.008). The mean amount allocated to the world, was significantly *higher* for those with high (M = 0.368, SD = 0.223) compared to low GC (M = 0.302, SD = 0.245, *p* = 0.001). Mean amount allocated to the world was also marginally higher for those with medium (M = 0.34, SD = 0.232) compared to low GC (*p* = 0.05). There was no significant difference between medium and high GC profiles at Time 1 in amounts allocated to the world at Time 2 (*p* = 0.15).

*Self-centrism scores* were calculated by subtracting amount allocated to the local group from amount reserved for self**.** There was a small significant main effect of GC (Time 1) on self-centrism in the MLPG (Time 2), (F(2, 621.75) = 8.33, *p* < 0.001, ω^2^ = 0.01). This became non-significant when controlling for demographics (age, gender, education and subjective SES). As seen in Fig. 5, mean self-centrism at Time 2 was significantly *lower* for those with high (M = 0.049, SD = 0.238) compared to low (M = 0.133, SD = 0.365, *p* = 0.003) and medium GC (M = 0.106, SD = 0.291, *p* = 0.002) at Time 1. There was no significant difference between low and medium GC profiles (*p* = 0.49).

*National-centrism* scores were calculated by subtracting amount allocated to world from amount allocated to local group. There were *no* significant differences between GC profiles (Time 1) in levels of national-centrism in the MLPG (Time 2), (F(2, 1456) = 2.63, *p* = 0.07).Interestingly, all groups donated more to world than to local group. This difference was largest for those with high GC; however, this did not reach significance without controls. This became significant when controlling for demographics (age, gender, education and subjective SES), where low GC (M = 0.13, SD = 0.36) were highest in national-centrism followed by medium (M = 11, SD = 0.29) and high GC (M = 0.05, SD = 0.24), (F(2, 1456) = 7.79, p < 0.001, η2 = 0.011).

*Global-centrism* scores were calculated by subtracting amount reserved for self from amount allocated to world. There was a small significant main effect of GC (Time 1) on global-centrism in the MLPG (Time 2), (F(2, 1456) = 8.39, *p* < 0.001, η^2^ = 0.011). As seen in Fig. 5, mean global-centrism at Time 2 was significantly higher for those with high (M = 0.03, SD = 0.394) compared to low (M = -0.113 SD = 0.469, *p* < 0.001) and medium GC (M = -0.043, SD = 0.429, *p* = 0.028) at Time 1. There was also significantly higher global-centrism exhibited by medium compared to low GC participants (*p* = 0.047). High GC participants allocated more to world while low and medium GC participants kept more for themselves on average.

**Figure 6:**
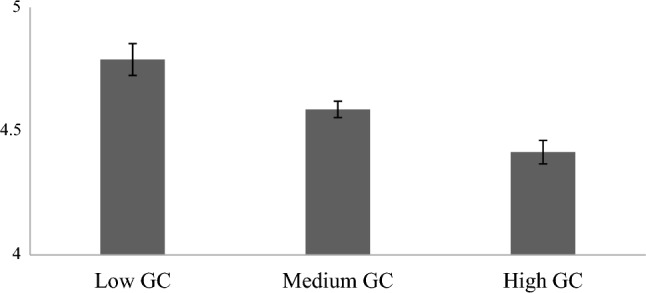
Earnings ($) across GC profiles in the Dictator (Experiment 1) and PD games (Experiment 2).

*Earnings* made by those who participated in both experiments were significantly different across GC profiles overall, indicated by a small main effect (F(2, 323.52) = 11.28, *p* < 0.001, ω^2^ = 0.02). As shown in Fig. 6, earnings for low GC were significantly higher than earnings made by medium (*p* = 0.016) and high GC profiles (*p* < 0.001). Earnings for medium GC were also significantly higher than earnings for high GC (*p* = 0.008).

## Superpower by GC interactions

We checked for any interactions between country and GC through a series of 2 (China, USA) X 3 (low, medium, high GC) ANOVAs to predict each of the behavioral outcomes.

In the first experiment, we found no significant interactions between country and GC profile in predicting any of the behavioral outcomes. In the second experiment, we did find a significant interaction between country and GC profile (Time 1) to predict donations to the *ingroup* in the PDG (Time 1), *F*(2, 1615) = 4.57, *p* = 0.01, η^2^ = 0.006. Simple effects analysis showed that in the USA, there were significant differences across all GC profiles with higher donations corresponding with higher GC. In China, there was no significant difference between medium and low GC profiles. There was a further significant interaction between country and GC (Time 1) to predict donations to the *outgroup* in the PDG (Time 1), *F*(2, 1615) = 6.14, *p* = 0.002, η^2^ = 0.008. Simple effects analysis showed the same pattern as before, where there were significant differences between all GC profiles in the USA with higher donations corresponding with higher GC, whereas there was no significant difference between low and medium GC in China. There was no significant interaction between country and GC at Time 1 to predict the *difference* of donations made to the ingroup and outgroup in the PDG at Time 1 (i.e., ingroup favouritism).

Also, in the second experiment, there was a significant interaction between country and GC (Time 1) to predict donations to the World in the multi-level public goods dilemma (at Time 2), *F*(2, 426) = 5.81, *p* = 0.003, η^2^ = 027. Simple effects analysis indicated that low GC participants donated significantly less to the world than medium and high GC participants in the USA, while there were no significant differences across GC profiles in China. Relatedly, there was a significant interaction to predict *global-centrism* in donations F(2, 426) = 4.33, *p* = 0.014, η^2^ = 02. As with the previous pattern, participants with low GC showed significantly lower global-centrism (donating less to the world than self) compared to other profiles in the USA, while there were no significant differences in China. There was also a significant interaction to predict *self-centrism* in donations, F(2, 426) = 5.9, *p* = 0.003, η^2^ = 0.027. Low GC participants showed significantly *more* self-centrism (donating more to self than local group) than the other two profiles in the United States, whereas there were no significant differences in China.

## Summary and discussion

Across six societies, the most robust findings were first that individuals high in global consciousness (GC) gave more to both ingroup and outgroup charities in an altruistic decision formatted as a dictator game; and they were less parochial in this task compared to those medium or low in GC. Second, those high in GC were more cooperative to both ingroup and outgroup members in a PDG (see also 49) and less parochial than those medium or low in GC. Longitudinal findings showed that those high in GC were less parochial than those medium or low in GC in a PDG three months after GC was first assessed. Similarly, those high in GC were less selfish in a MLPG and contributed more to the public good than those medium or low in GC. GC is a win–win benefitting *both* the “nation-state” *and* the world in every experimental test posed here.

These results are promising, as cross-cultural evidence for the behavioral consequences of GC was found, encompassing citizens of both the highly individualistic USA and the more collectivistic China, and several other Asian societies. The results for GC were the least compelling for Hong Kong, a Special Administrative Region of the People’s Republic of China, where identification with the local administrative unit and the larger nation is uneven^29^. Results for Hong Kong showed trends similar to other societies, but did not reach significance, demonstrating that the behavioral consequences of GC can be sensitive to macro-level political context/turmoil^50^. This attests to the dynamism of GC as a construct embedded in the broader societal and cultural context.

Individualism versus collectivism has long been used to reflect cultural values of prioritizing individual pursuits versus group cohesion^30^. Globalization has made cultures increasingly interconnected. Many cultures are becoming more individualistic^31^. GC transcends the self versus group continuum and extends one’s ingroup beyond national culture. Its behavioral consequences have important implications for cultural change over time. Interestingly, the results of the superpower countries suggest that charitable giving may be a more sensitive domain as an outcome behavior for GC among citizens of the USA than China. China is still a developing country economically, and charitable giving may be less firmly inscribed as a domain of moral behavior than in the USA.

Future research could usefully augment the experimental results reported here through survey methods examining correlates of GC with such real-life measures such as charitable giving or environmental conservation/activism. It could also explore the advantages and disadvantages of the latent profile approach, which focuses attention on the PERSON by creating a profile that aggregates across measures of GC, versus a more traditional factor analytical approach, which focuses attention on VARIABLES, by analyzing different aspects of GC as correlated but separate measures. We acknowledge that although significant, the effects found were smaller than might be expected theoretically, especially in the analyses using Cosmopolitan Orientation, Global Orientations and Identification With All Humanity scales as separate predictors (Tables S12, S13). As such, it is important for future research to follow up on these exploratory analyses to investigate what may be driving the consistently significant but relatively small effects across societies and contexts.

Theorists have long recognized individual differences can be drivers of cultural change in small groups during evolution^32,33^, especially during critical junctures of societal change^49^. The one-shot social dilemmas operationalized here cannot fully capture the promise of GC as an agent-based feature that may assist humanity in shaping a viable collective future in the real social dilemmas facing it. In one-shot social dilemmas, GC is costly. However, it is well-known that through repeated interactions, cooperative individuals can thrive by forming alliances with other cooperators^34^. Reputation also matters^35^. If highly successful individuals (e.g., beneficiaries of the global economy) were seen as high in GC, this could drive cultural evolution, through imitative and reputational processes, that may in certain situations be strong enough to overcome tendencies to conform^33^ to the status quo of national parochialism. If the world’s economy continues to become more global, GC is likely to grow because it is adaptive to market conditions, just as members of small-scale societies modified their behaviors in economic exchange as their societies became more market integrated^36^. Such is the promise of global consciousness today, and for future generations.

## Methods

This research and procedure were conducted under Ethics Notification Number: 4000022749, Global Consciousness in East Asia and the United States of America, that was assessed as Low Risk by the Massey University Human Ethics Committee. All methods were performed in accordance with the relevant guidelines and regulations.

### Participants

We partnered with Toluna, an international market research company based in Australia to collect data from six cultures. Experiment one, wave 1 (from July 2 to July 16, 2020) involved 6138 participants, with 1449 participants retained in wave 2 (administered from October 5 to November 8, 2020). Experiment two, wave 1 (from March 28 to May 7, 2021) involved 4925 participants, with 1459 retained in wave 2 (from July 5 to August 8, 2021) (for details, see SI Table S1). A clear statement of informed consent was provided to all participants, who could choose whether to proceed with the study or not. Table 1 shows a summary of the demographics of each wave of the studies, indicating that all samples were around the mid-point in terms of self-reported status, age and relatively well educated with an even split across genders. A breakdown of demographics by country sample (see supplementary materials Tables S1 and S2) further shows that the samples were comparable in terms of key demographics.

**Table 1:** Demographics of the sample from both waves of each study.

	Age, M (SD)	Gender (% female)	Education, M (SD)	Social status, M (SD)
Study 1, Wave 1	41.98 (12.86)	51.1%	4.61 (1.01)	5.48 (1.88)
Study 1, Wave 2	43.4 (12.17)	49.7%	4.64 (.96)	5.33 (1.67)
Study 2, Wave 1	39.94 (13.51)	50.2%	4.95 (1.26)	5.71 (1.92)
Study 2, Wave 2	43.8 (13.33)	52.6%	4.91 (1.18)	5.33 (1.84)

A G*Power analysis indicated that we would need a minimum sample size of 321 in a one-way ANOVA (3 levels) to detect a small effect size as significant (*p* < 0.05) with 90% power. All country/society samples from the first wave of both studies met this requirement. In both studies, 5 of the 6 country/society samples from the second wave were below this requirement. However, given that our main analyses combined the samples of all countries, both waves of each study were sufficiently powered to detect an *overall* small effect as significant. To detect a small (0.2) interaction effect with 90% power in a 2 × 3 between-subjects ANOVA (alpha = 0.05), G*Power indicated that we would need at least 54 participants in each group (*N* = 320). The combined US and China samples (across both waves of each study) were sufficiently powered for detecting a small interaction effect.

### Procedure and experimental design

Participants responded to an online survey. The initial version of the survey was written in English, and then each survey was translated (and back-translated) by experts in the language (or the committee method was used). The same procedure was used across all samples and surveys. First, participants were asked to agree to an informed consent form. Next, they read instructions that explained the behavioral decision-making task required for the particular wave of the given experiment (described previously: dictator game in Experiment 1, wave 1, prisoner’s dilemma in Experiment 1, wave 2, prisoner’s dilemma in Experiment 2, wave 1, and multi-level public goods game in Experiment 2, wave 2). There was hence only one type of behavioral decision-making task in each wave: if these involved multiple trials with experimental manipulations, the trials were counter-balanced in terms of order. After this, participants completed a number of measures, including the ones used in the research reported here to measure GC.

### Materials and measures

#### Global orientations

We selected 20 items from the Global Orientations (GO) scale^20^ in both waves of Experiment 1, which included items such as “One should actively involve himself or herself in a multicultural environment” (for the first factor, multicultural acquisition), and “I find living in a multicultural environment very stressful” (for the second factor, ethnic protection). All responses were measured on a 7-point scale. A 2-factor model of Global Orientations was tested in line with the original model. In Experiment 2, in both waves we only included the 16 items from the 2-factor model used in Experiment 1 (see S1 Appendix S1 for final items used).

#### Cosmopolitan orientation

All 15 items from the Cosmopolitan Orientation (CO) scale^15^ were included in both waves of both studies, with items such as, “I want to help the unfortunate ones even if they are from other countries” (for the first factor, global prosociality), “I want to travel to experience many different cultures” (for the second factor, cultural openness), and “I respect cultural differences” (for the third factor, respect for cultural diversity). All responses were measured on a 6-point scale. We tested a 3-factor model, in line with the original model.

#### Identification with all humanity

We selected 8 items from the Identification With all Humanity (IWAH) scale^16^ for both waves in Experiment 1, with items such as “How often do you use the word “we” to refer to the following groups of people? (People all over the world)” (for the first factor, bond) and “How much would you say you care (feel upset, want to help) when bad things happen to (People anywhere in the world)” (for the second factor, concern). All responses were measured on a 5-point scale. Both a 1-factor and 2-factor model were tested in accord with recent modification to the model^19^. The 2-factor model had superior fit, and was used subsequently. Seven of the 8 items were included in both waves of Experiment 2 (see SI Appendix S1, for final items used).

### Analyses

In Experiment 1, we tested the measurement invariance of a 2-factor model of GO, a 3-factor model of CO, and a 2-factor model of IWAH across both waves, in all 6 societies. For GO, we selected items with high factor loadings resulting in a 2-factor model with 10 positively worded items and 6 negatively worded items. After item deletions, there was good model fit for both configural and metric invariance for all scales across both waves. The exact same items and measurement models achieved configural and metric invariance for all scales in Experiment 2 (both waves) as well (details in SI, Tables S3–S8).

To identify the optimal latent profile solution for the two factors of GO, three factors of CO, and two factors of IWAH described above, we examined a range of solutions by using the Vong-Lo-Mendell-Rubin (VLMR) testing procedure as well as the Information Criteria (Akaike Information Criterion [AIC] and adjusted Bayesian Information Criterion [aBIC]). Using these criteria, we identified a 3-profile model as the optimal solution across both waves of the two experiments (details in SI, Tables S9 and S10, Figs. S1–S6), compared to alternative models ranging from 2 to 5 profiles.

Finally, to examine stability and change in the Global Consciousness (GC) profiles, a latent transition analysis was conducted with those who participated in wave 1 of both Experiment 1 and Experiment 2 (*N* = 847). The proportions of each profile and transition probability are presented in SI Table S11. Over the course of roughly 8 months, more than 80% of participants stayed in the same GC profile, indicating the GC profiles were relatively stable.

### Supplementary Information

Supplementary Information.

## Data Availability

All data, code, and materials used in the analysis are available on request from JHL at j.h.liu@massey.ac.nz. Data can be downloaded directly from https://osf.io/6yebu/.
