# Supplementary material for: Behavioral evidence for global consciousness transcending national parochialism
Source: Sci Rep. 2023 Dec 4;13:21413. doi: 10.1038/s41598-023-47333-z (PMC10695953; doi:10.1038/s41598-023-47333-z)
Supplement: Supplementary file 1 — Supplementary Information. [file 41598_2023_47333_MOESM1_ESM.docx]

Supplementary Materials for

**Behavioral Evidence for Global Consciousness Transcending National Parochialism**

**This file includes:**

Supplementary Text

Figs. S1 to S7

Tables S1 to S14

**Materials and Measures**

***Cosmopolitan Orientation***

All 15 items from the Cosmopolitan Orientation (CO) scale^15^ were included in both studies, with items such as, “I want to help the unfortunate ones even if they are from other countries” (for the first factor, global prosociality), “I want to travel to experience many different cultures” (for the second factor, cultural openness), and “I respect cultural differences” (for the third factor, respect for cultural diversity). All responses were measured on a 6-point scale. We tested a 3-factor model, in line with the original model.

***Global Orientations***

We selected 20 items from the Global Orientations (GO) scale^20^ in Experiment 1, which included items such as “One should actively involve himself or herself in a multicultural environment” (for the first factor, multicultural acquisition), and “I find living in a multicultural environment very stressful” (for the second factor, ethnic protection). All responses were measured on a 7-point scale. A 2-factor model of Global Orientations was tested in line with the original model. In Experiment 2, we only included the 16 items from the resulting model in Experiment 1 (see Appendix for final items used).

***Identification With All Humanity***

We selected 8 items from the Identification With all Humanity (IWAH) scale^16^ in Experiment 1, with items such as “How often do you use the word “we” to refer to the following groups of people? (People all over the world)” (for the first factor, bond) and “How much would you say you care (feel upset, want to help) when bad things happen to (People anywhere in the world)” (for the second factor, concern for humanity). All responses were measured on a 5-point scale. Both a 1-factor and 2-factor model were tested in accord with recent modifications to the model^19^. The 2-factor model had superior fit, and so is used subsequently. Seven of the 8 items were included in Experiment 2 (see Appendix for final items used).

**Demographic Characteristics**

***Experiment 1***

**Table S1.** Demographics of each society or country sample in Experiment 1 across waves

| *Wave 1* | Age M (SD) | Gender (% Female) | Education M (SD) | Social Status M (SD) |
| --- | --- | --- | --- | --- |
| China (N = 1017) | 37.25 (10.32) | 51.6 | 4.8 (.71) | 5.42 (1.55) |
| Hong Kong (N = 1000) | 40.82 (10.32) | 58.1 | 4.33 (1.08) | 4.74 (1.69) |
| Malaysia (N = 998) | 37.36 (10.18) | 48.8 | 4.38 (1.08) | 5.74 (1.83) |
| Singapore (N = 999) | 41.6 (11.32) | 42.1 | 4.78 (.99) | 6.09 (1.86) |
| Taiwan (N = 1000) | 39.44 (10.25) | 52.1 | 4.75 (.97) | 4.95 (1.69) |
| United States (N = 1124) | 54 (14.91) | 53.6 | 4.63 (1.05) | 5.87 (2.15) |

| *Wave 2* | Age M (SD) | Gender (% Female) | Education M (SD) | Social Status M (SD) |
| --- | --- | --- | --- | --- |
| China (N = 220) | 36.4 (8.98) | 51.1 | 4.83 (.61) | 5.53 (1.56) |
| Hong Kong (N = 337) | 42.35 (9.99) | 53.4 | 4.47 (1.06) | 4.95 (1.71) |
| Malaysia (N = 188) | 40.17 (11.0) | 47.3 | 4.41 (1.02) | 5.71 (1.71) |
| Singapore (N = 205) | 44.44 (12.09) | 35.6 | 4.77 (.92) | 5.84 (1.61) |
| Taiwan (N = 314) | 42.18 (10.27) | 48.1 | 4.87 (.88) | 5.19 (1.64) |
| United States (N = 185) | 56.85 (12.96) | 61.6 | 4.43 (1.08) | 5.24 (1.84) |

***Experiment 2***

**Table S2.** Demographics of each society or country sample in Experiment 2 across waves

| *Wave 1* | Age M (SD) | Gender (% Female) | Education M (SD) | Social Status M (SD) |
| --- | --- | --- | --- | --- |
| China (N = 758) | 41.16 (13.52) | 50.1 | 4.7 (.75) | 5.59 (1.62) |
| Hong Kong (N = 798) | 39.77 (12.31) | 50.1 | 4.44 (1.04) | 4.97 (1.74) |
| Malaysia (N = 864) | 36.22 (12.63) | 49.2 | 4.42 (.97) | 5.97 (1.75) |
| Singapore (N = 824) | 39.93 (13.48) | 49.8 | 6.69 (1.07) | 6.27 (1.89) |
| Taiwan (N = 823) | 40.94 (13.34) | 49.8 | 4.72 (.92) | 5.14 (1.69) |
| United States (N = 858) | 41.78 (14.88) | 52.2 | 4.75 (1.07) | 6.7 (2.3) |

| *Wave 2* | Age M (SD) | Gender (% Female) | Education M (SD) | Social Status M (SD) |
| --- | --- | --- | --- | --- |
| China (N = 231) | 40.01 (11.23) | 50.6 | 4.67 (.72) | 5.64 (1.71) |
| Hong Kong (N = 339) | 42.19 (1.73) | 51.6 | 4.5 (1.02) | 4.9 (1.79) |
| Malaysia (N = 212) | 39.77 (12.85) | 49.1 | 4.47 (.92) | 5.5 (1.77) |
| Singapore (N = 231) | 45.67 (13.62) | 51.5 | 6.57 (1.03) | 5.73 (1.82) |
| Taiwan (N = 250) | 42.92 (11.4) | 52.3 | 4.77 (.87) | 5.04 (1.74) |
| United States (N = 196) | 54.32 (14.9) | 62.2 | 4.57 (.99) | 5.42 (2.09) |

**Measurement Invariance**

***Experiment 1***

We tested the measurement invariance of a 2-factor model of GO, a 3-factor model of CO, and a 2-factor model of IWAH across both waves, in all 6 societies. For GO, we cut a few items with unsatisfactory factor loadings resulting in a 2-factor model with 10 positively worded items and 6 negatively worded items. As can be seen in Tables S1-S3, there was good model fit for both configural and metric invariance across both waves.

**Table S3.** Measurement invariance testing for the 2-factor model of GOS.

| Model | χ^2^ (df) | RMSEA | CFI | TLI | SRMR | ΔRMSEA | ΔCFI |
| --- | --- | --- | --- | --- | --- | --- | --- |
| *Wave 1* |  |  |  |  |  |  |  |
| Configural | 3196.605 (618) | .064 [.062, .066] | .919 | .906 | .054 |  |  |
| **Metric** | **3491.484 (688)** | **.063 [.061, .065]** | **.912** | **.908** | **.069** | **-.001** | **-.007** |
| Scalar | 6395.493 (753) | .086 [.084, .088] | .824 | .832 | .096 | -.088 | -.076 |
| *Wave 2* |  |  |  |  |  |  |  |
| Configural A | 1351.106 (618) | .070 [.065, .075] | .912 | .897 | .062 |  |  |
| Configural B | 1261.034 (612) | .066 [.061, .071] | .922 | .908 | .061 |  |  |
| **Metric B** | **1367.133 (682)** | **.064 [.060, .069]** | **.918** | **.913** | **.087** | **-.002** | **-.004** |
| Scalar B | 2326.658 (752) | .093 [.089, .097] | .811 | .819 | .110 | .029 | -.107 |
| *Note.* In Configural B model, the residual correlation between GOS17 and GOS18 was allowed to be freely estimated. | | | | | | | |

**Table S4.** Measurement invariance testing for the 3-factor model of COS.

| Model | χ^2^ (df) | RMSEA | CFI | TLI | SRMR | ΔRMSEA | ΔCFI |
| --- | --- | --- | --- | --- | --- | --- | --- |
| *Wave 1* |  |  |  |  |  |  |  |
| Configural | 2569.947 (522) | .062 [.060, .064] | .944 | .933 | .044 |  |  |
| **Metric** | **2890.245 (582)** | **.062 [.060, .065]** | **.937** | **.932** | **.065** | **.000** | **-.007** |
| Scalar | 4489.100 (642) | .077 [.074, .079] | .896 | .898 | .080 | .015 | -.041 |
| *Wave 2* |  |  |  |  |  |  |  |
| Configural | 1086.124 (522) | .067 [.061, .072] | .940 | .927 | .053 |  |  |
| **Metric** | **1199.071 (582)** | **.066 [.061, .072]** | **.934** | **.929** | **.077** | **-.001** | **-.006** |
| Scalar | 1755.475 (642) | .085 [.080, .089] | .881 | .883 | .093 | .019 | .053 |

**Table S5.** Measurement invariance testing for the 2-factor model of IWAH.

| Model | χ^2^ (df) | RMSEA | CFI | TLI | SRMR | ΔRMSEA | ΔCFI |
| --- | --- | --- | --- | --- | --- | --- | --- |
| *Wave 1* |  |  |  |  |  |  |  |
| Configural | 515.991 (78) | .074 [.068, .080] | .973 | .956 | .031 |  |  |
| **Metric** | **589.399 (103)** | **.068 [.063, .073]** | **.970** | **.963** | **.045** | **-.006** | **-.003** |
| Scalar | 1129.160 (128) | .087 [.083, .092] | .937 | .938 | .066 | .019 | -.033 |
| *Wave 2* |  |  |  |  |  |  |  |
| Configural A | 248.608 (78) | .095 [.082, .109] | .958 | .932 | .045 |  |  |
| Configural B | 184.030 (72) | .080 [.066, .095] | .972 | .952 | .037 |  |  |
| **Metric B** | 221.097 (97) | .073 [.060, .085] | .969 | .960 | .059 | -.007 | -.003 |
| Scalar B | 497.269 (122) | .113 [.103, .123] | .907 | .904 | .095 | .040 | -.062 |
| *Note.* In Configural B model, the residual correlation between IWAH6 and IWAH7 was allowed to be freely estimated. | | | | | | | |

***Experiment 2***

We tested the measurement invariance of a 2-factor model of GO, a 3-factor model of CO, and a 2-factor model of IWAH as the same in Experiment 1, across both waves, in all 6 societies. As can be seen in Tables S6-S8, there was good model fit for both configural and metric invariance across both waves.

**Table S6.** Measurement invariance testing for the 2-factor model of GOS.

| Model | χ^2^ (df) | RMSEA | CFI | TLI | SRMR | ΔRMSEA | ΔCFI |
| --- | --- | --- | --- | --- | --- | --- | --- |
| *Wave 1* |  |  |  |  |  |  |  |
| Configural | 3196.605 (618) | .064 [.062, .066] | .919 | .906 | .054 |  |  |
| **Metric** | **3491.484 (688)** | **.063 [.061, .065]** | **.912** | **.908** | **.069** | **-.001** | **-.007** |
| Scalar | 6395.493 (753) | .086 [.084, .088] | .824 | .832 | .096 | .023 | -.076 |
| *Wave 2* |  |  |  |  |  |  |  |
| Configural | 1260.904 (618) | .065 [.060, .071] | .919 | .906 | .065 |  |  |
| **Metric** | **1379.779 (688)** | **.064 [.059, .069]** | **.913** | **.909** | **.089** | **-.001** | **-.006** |
| Scalar | 2111.237 (758) | .086 [.081, .090] | .830 | .838 | .115 | .022 | -.075 |

**Table S7.** Measurement invariance testing for the 3-factor model of COS.

| Model | χ^2^ (df) | RMSEA | CFI | TLI | SRMR | ΔRMSEA | ΔCFI |
| --- | --- | --- | --- | --- | --- | --- | --- |
| *Wave 1* |  |  |  |  |  |  |  |
| Configural | 2569.947 (522) | .062 [.060, .064] | .944 | .933 | .044 |  |  |
| **Metric** | **2890.245 (582)** | **.062 [.060, .065]** | **.937** | **.932** | **.065** | **.000** | **-.007** |
| Scalar | 4489.100 (642) | .077 [.074, .079] | .896 | .898 | .080 | .015 | -.041 |
| *Wave 2* |  |  |  |  |  |  |  |
| Configural | 988.163 (522) | .061 [.055, .066] | .951 | .941 | .048 |  |  |
| **Metric** | **1121.549 (582)** | **.062 [.056, .067]** | **.944** | **.939** | **.081** | **.001** | **-.007** |
| Scalar | 1524.885 (642) | .075 [.070, .080] | .908 | .910 | .095 | .013 | -.036 |

**Table S8.** Measurement invariance testing for the 2-factor model of IWAH.

| Model | χ^2^ (df) | RMSEA | CFI | TLI | SRMR | ΔRMSEA | ΔCFI |
| --- | --- | --- | --- | --- | --- | --- | --- |
| *Wave 1* |  |  |  |  |  |  |  |
| Configural | 515.991 (78) | .074 [.068, .080] | .973 | .956 | .031 |  |  |
| **Metric** | **589.399 (103)** | **.068 [.063, .073]** | **.970** | **.963** | **.045** | **-.006** | **-.003** |
| Scalar | 1129.160 (128) | .087 [.083, .092] | .937 | .938 | .066 | .019 | -.033 |
| *Wave 2* |  |  |  |  |  |  |  |
| Configural | 217.920 (78) | .086 [.073, .100] | .963 | .940 | .043 |  |  |
| **Metric** | **250.891 (103)** | **.077 [.065, .089]** | **.961** | **.952** | **.058** | **-.009** | **-.002** |
| Scalar | 384.614 (128) | .091 [.080, .101] | .932 | .933 | .069 | .014 | -.029 |

**Global Consciousness (GC) Profiles:**

To identify the optimal latent profile solution, we examined a range of solutions by using the Vong-Lo-Mendell-Rubin (VLMR) testing procedure as well as the Information Criteria (Akaike Information Criterion [AIC] and adjusted Bayesian Information Criterion [aBIC]). Lower values of the Information Criteria suggest a better fit. The VLMR statistics compares a *k* profile solution with a *k*−1 profile solution, and a significant *p* value suggests that the *k* profile has a better fit than does the *k*−1 profile. In addition, entropy was used as a supplementary criterion. Higher values indicate a clearer separation of profiles (range = 0–1, and entropy > .80 is typically seen as a cut-off point). We also assessed the substantive contribution and interpretability of the profiles identified.

***Experiment 1***

The Information Criteria and the VLMR statistics for solutions ranging from 2 to 5 profiles are presented in Table S9. The Information Criteria and the VLMR test suggested that the 3-profile model provides a good solution (entropy = .807 in Wave 1, .794 in Wave 2), and this solution was clearly interpretable. Thus, we identified the 3-profile model as the optimal solution across both waves.

The 3 identified global consciousness (GC) profiles (wave 1) are presented in Figure S1. The largest profile (48.3%, *n* = 2963)—*medium GC*—was characterized by moderate levels of global consciousness across all factors of GC. The second-largest profile (38.2%, *n* = 2344)—*high GC*—was characterized by high levels of GC across 6 factors, with a moderate level of Ethnic Protection. The smallest profile (13.5%, *n* = 831)—*low GC*—was characterized by low levels across all factors, except Ethnic Protection and Identification, which were low to moderate. This same order of profiles was evident in wave 2. We note that participants across all three profiles had similar scores on Ethnic Protection. This may be because Ethnic Protection is the only GC indicator that is defensive, reflecting national consciousness rather than being concerned with the benefits of a globalized environment, whereas all other 6 GC indicators are proactive toward globalization. Future research could drop this factor from GC as it does not correlate strongly with the other factors.

**Table S9.** Information Criteria and the VLMR statistics for the different latent profile solutions, based on standardized scores

|  | AIC | aBIC | VLMR (p) | Entropy |
| --- | --- | --- | --- | --- |
| *Wave 1* |  |  |  |  |
| 2 profiles | 111788.994 | 111866.974 | 10094.141 (< .001) | .816 |
| **3 profiles** | **108751.603** | **108857.939** | **3053.391 (< .001)** | **.807** |
| 4 profiles | 107270.563 | 107405.255 | 1497.040 (.0009) | .803 |
| 5 profiles | 106241.799 | 106404.847 | 1044.764 (< .001) | .810 |
| *Wave 2* |  |  |  |  |
| 2 profiles | 26270.593 | 26316.836 | 2515.908 (< .001) | .824 |
| **3 profiles** | **25555.813** | **25618.871** | **730.780 (.0088)** | **.794** |
| 4 profiles | 25192.908 | 25272.782 | 378.905 (.0103) | .838 |
| 5 profiles | 24949.812 | 25046.502 | 259.096 (< .001) | .844 |
| *Note.* N (Wave 1) = 6138; N (Wave 2) = 1449; AIC – Akaike Information Criterion; aBIC – adjusted Bayesian Information Criterion; VLMR – Vong-Lo-Mendell-Rubin statistic (*p* value). | | | |  |

**Figure S1.**

The identified latent global consciousness profiles and mean component levels across the 7 factors of global orientations, cosmopolitan orientation, and IWAH (Exp 1, wave 1)

*
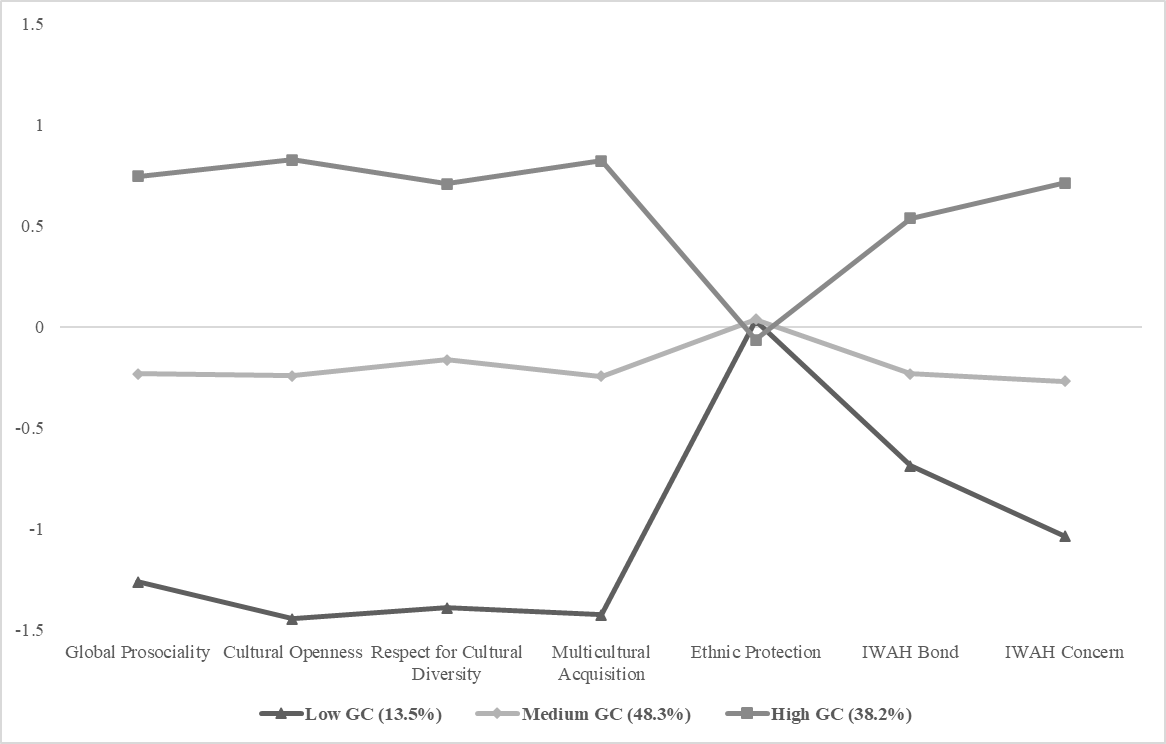
*

**Figure S2.**

Alternative solution of 4 latent global consciousness profiles and mean component levels across the 7 factors of global orientations, cosmopolitan orientation, and IWAH (Exp 1, wave 1)

**Figure S3.**

Alternative solution of 5 latent global consciousness profiles and mean component levels across the 7 factors of global orientations, cosmopolitan orientation, and IWAH (Exp 1, wave 1)

***Experiment 2***

The Information Criteria and the VLMR statistics for solutions ranging from 2 to 5 profiles are presented in Table S8. The Information Criteria and the VLMR test suggested that the 3-profile model provided a good solution (entropy = .813 in Wave 1, .862 in Wave 2), and this solution was clearly interpretable. Thus, we identified the 3-profile model as the optimal solution across both waves.

The 3 identified global consciousness profiles (wave 1) are presented in Figure S2. The largest profile (49.4%, *n* = 2433)—*medium GC*—was characterized by moderate levels of global consciousness across all factors of GC. The second-largest profile (32.1%, *n* = 1579)—*high GC*—was characterized by high levels of GC across 6 factors, with a moderate level of Ethnic Protection. The smallest profile (18.5%, *n* = 913)—*low GC*—was characterized by low levels across all factors, except Ethnic Protection and Identification, which were low to moderate. The same order of profiles was evident in Wave 2.

**Table S10.** Information Criteria and the VLMR statistics for the different latent profile solutions, based on standardized scores

|  | AIC | aBIC | VLMR (p) | Entropy |
| --- | --- | --- | --- | --- |
| *Wave 1* |  |  |  |  |
| 2 profiles | 88712.897 | 88786.035 | 8992.141( <.001) | .827 |
| **3 profiles** | **85728.758** | **85828.491** | **2956.669 (< .05)** | **.813** |
| 4 profiles | 83814.612 | 83940.94 | 1287.627 (<.001) | .843 |
| 5 profiles | 82530.471 | 82683.395 | 1281.303 (.11) | .848 |
| *Wave 2* |  |  |  |  |
| 2 profiles | 26815.869 | 26862.236 | 2168.488 (<.001) | .796 |
| **3 profiles** | **25893.349** | **25956.614** | **922.665 (<.001)** | **.862** |
| 4 profiles | 25497.748 | 25577.883 | 404.682 (.218) | .809 |
| 5 profiles | 25226.203 | 25323.209 | 205.533 (0.672) | .831 |
| N (Wave 1) = 4925, N (Wave 2) = 1459; AIC – Akaike Information Criterion; aBIC – adjusted Bayesian Information Criterion; VLMR – Vong-Lo-Mendell-Rubin statistic (*p* value). | | | |  |

**Figure S4.**

The identified latent global consciousness profiles and component mean levels across the 7 factors of global orientations, cosmopolitan orientation and IWAH (Experiment 2, Wave 1)

**Figure S5.**

Alternative solution of 4 latent global consciousness profiles and mean component levels across the 7 factors of global orientations, cosmopolitan orientation, and IWAH (Exp 2, wave 1)

**Figure S6.**

Alternative solution of 5 latent global consciousness profiles and mean component levels across the 7 factors of global orientations, cosmopolitan orientation, and IWAH (Exp 2, wave 1)

**Cross-study Latent Transition Analysis**

To examine the stability and change of the Global Consciousness profiles, a latent transition analysis was conducted with those overlapping participants who participated in wave 1 of both Experiment 1 and Experiment 2 (*N* = 847). The proportions of each profile and transition probability are presented in Table S9. Over the course of roughly 8 months, more than 80% of participants stayed in the same GC profile, indicating the GC profiles were relatively stable between the 2 time points of the two studies. This level of test-retest stability is in line with that of widely used measures of adult personality across the lifespan^5^ .

**Table S11.** Latent Transition Analysis of Participants who completed Experiment 1 wave 1 and Experiment 2 wave 1

|  | Low GC (%) | Medium GC (%) | High GC (%) |
| --- | --- | --- | --- |
| **Proportions of each profile** | | | |
| Experiment 1 wave 1 | 17.9 | 49.8 | 32.2 |
| Experiment 2 wave 1 | 21.0 | 46.6 | 32.3 |
| **Transition probabilities (rows for Experiment 1 and columns for Experiment 2)** | | | |
| Low GC | 80.1 | 17.3 | 2.7 |
| Medium GC | 10.2 | 80.5 | 9.3 |
| High GC | 3.2 | 12.8 | 84.0 |

**Overall Earnings in Experiments 1 and 2**

Finally, overall earnings were significantly different across GC profiles in both Experiment 1 (F(2, 1438) = 10.4, *p* < .001, η^2^ = .013) and Experiment 2 (F(2, 1452) = 7.37, *p* < .001, η^2^ = .018). As shown in Figure S3, in Experiment 1, low GC earnings (M = 4.48, SD = .75) were significantly higher than both medium (M = 4.31, SD = .72, *p*  = .016) and high GC earnings (M = 4.21, SD = .76, *p* < .001), while medium GC earnings were significantly higher than earnings for those with high GC (*p* = .037). In Experiment 2, low GC participants (M = 5.21, SD = .57) earned significantly more than medium (M = 5.06, SD = .51, *p* < .001) and high GC participants (M = 4.99, SD = .52, *p* < .001), though the difference between medium and high GC earnings was not significant (*p*  = .093). There is without doubt a short-term cost to high GC in terms of financial earnings in one-shot social dilemmas.

**Figure S7.**

Overall earnings made ($) across GC profiles

*Note.* Experiment 1 includes earnings from the Dictator (W1) and Prisoner’s Dilemma games (W2); Experiment 2 includes earnings from the Prisoner’s Dilemma (W1) and Multi-level Goods games (W2)

**Table S12.** Linear regression models using 7 GC indicators as separate independent variables to predict national parochialism in Experiment 1, controlling for demographic covariates (age, gender, education, and subjective SES).

| Independent Variables | *National Parochialism in charity giving (N = 6109)* | | |  | *National Parochialism in the Prison’s dilemma game (N = 1446)* | | |
| --- | --- | --- | --- | --- | --- | --- | --- |
|  | *B* | *SE* | *p* |  | *B* | *SE* | *p* |
| Global Prosociality | -0.001 | 0.002 | .51 |  | -0.004 | 0.004 | .278 |
| Cultural Openness | -0.008 | 0.002 | <.001 |  | -0.014 | 0.003 | <.001 |
| Respect for Cultural Diversity | 0.001 | 0.003 | .781 |  | -0.009 | 0.005 | .053 |
| Multicultural Acquisition | -0.007 | 0.002 | .007 |  | -0.008 | 0.004 | .062 |
| Ethnic Protection | 0.000 | 0.002 | .830 |  | -0.001 | 0.003 | .874 |
| IWAH Bond | -0.015 | 0.002 | <.001 |  | -0.011 | 0.004 | .009 |
| IWAH Concern | -0.1 | 0.002 | <.001 |  | -0.011 | 0.004 | .005 |

**Table S13.** Linear regression models using 7 GC indicators as separate independent variables to predict parochial generosity in Experiment 2, controlling for demographic covariates (age, gender, education, and subjective SES)..

| Independent Variables | *Parochial Generosity*  *(N = 4907)* | | |  | *National-centrism*  *(N = 1456)* | | |
| --- | --- | --- | --- | --- | --- | --- | --- |
|  | *B* | *SE* | *p* |  | *B* | *SE* | *p* |
| Global Prosociality | -0.012 | 0.002 | <.001 |  | -0.066 | 0.009 | <.001 |
| Cultural Openness | -0.015 | 0.002 | <.001 |  | -0.018 | 0.008 | .028 |
| Respect for Cultural Diversity | 0.002 | 0.003 | .559 |  | -0.05 | 0.011 | <.001 |
| Multicultural Acquisition | -0.008 | 0.003 | .001 |  | -0.026 | 0.009 | .006 |
| Ethnic Protection | 0.000 | 0.002 | .825 |  | -0.02 | 0.007 | .006 |
| IWAH Bond | -0.018 | 0.002 | <.001 |  | -0.013 | 0.01 | .186 |
| IWAH Concern | -0.018 | 0.002 | <.001 |  | -0.053 | 0.009 | <.001 |

|  | 1 | 2 | 3 | 4 | 5 | 6 | 7 | 8 | 9 |
| --- | --- | --- | --- | --- | --- | --- | --- | --- | --- |
| 1. Global Prosociality |  |  |  |  |  |  |  |  |  |
| 2. Cultural Openness | .577** |  |  |  |  |  |  |  |  |
| 3. Respect for Cultural Diversity | .593** | .614** |  |  |  |  |  |  |  |
| 4. Multicultural Acquisition | .550** | .744** | .635** |  |  |  |  |  |  |
| 5. Ethnic Protection | -0.010 | -.041** | -.054** | -.005 |  |  |  |  |  |
| 6. IWAH Bond | .389** | .390** | .229** | .412** | .072** |  |  |  |  |
| 7. IWAH Concern | .604** | .445** | .426** | .496** | -.060** | .626** |  |  |  |
| 8. English proficiency | .206** | .356** | .169** | .321** | -.175** | .288** | .319** |  |  |
| 9. Chinese proficiency | -.064** | .052* | .012 | -.016 | .114** | .051* | -.063** | .164** |  |

**Table S14.** Correlations between GC indicators and language proficiency in Experiment 1.

**Table S15.** Correlations between GC indicators and language proficiency in Experiment 2.

|  | 1 | 2 | 3 | 4 | 5 | 6 | 7 | 8 | 9 | 10 |
| --- | --- | --- | --- | --- | --- | --- | --- | --- | --- | --- |
| 1. Global Prosociality |  |  |  |  |  |  |  |  |  |  |
| 2. Cultural Openness | .631** |  |  |  |  |  |  |  |  |  |
| 3. Respect for Cultural Diversity | .624** | .623** |  |  |  |  |  |  |  |  |
| 4. Multicultural Acquisition | .649** | .762** | .691** |  |  |  |  |  |  |  |
| 5. Ethnic Protection | .087** | .070** | .038** | .149** |  |  |  |  |  |  |
| 6. IWAH Bond | .392** | .387** | .190** | .346** | .218** |  |  |  |  |  |
| 7. IWAH Concern | .647** | .500** | .438** | .500** | .071** | .613** |  |  |  |  |
| 8. English proficiency | .181** | .334** | .109** | .211** | -.101** | .271** | .311** |  |  |  |
| 9. Chinese proficiency | -.099** | -.031 | -.037 | -.086** | .073** | 0.040 | -.047 | .194** |  |  |
| 10. Number of languages | .034* | .165** | .033* | .113** | -.037* | .074** | .056** | .359** | .242** |  |

**Appendix**

1. Cosmopolitan Orientation Scale (COS) Items Used

Global Prosociality

1. I would serve the world community by helping human beings.

2. I get upset when people do not want to offer help when those in need are foreigners.

3. I want to play my part to help make the world a better place for all.

4. When people from other countries are in need, I will help them to the best of my abilities.

5. I want to help the unfortunate ones even if they are from other countries.

Cultural Openness

6. I am willing to study or work abroad in another culture.

7. I am open to living in a different culture.

8. I enjoy learning more about different cultures in the world.

9. I want to travel to experience many different cultures.

10. It is exciting to immerse in a foreign culture.

Respect for Cultural Diversity

11. I respect cultural differences.

12. It is important to preserve the authenticity of native cultures.

13. I embrace cultural diversity.

14. We should celebrate cultural differences.

15. I am against having one dominating culture.

2. Global Orientations (GO) Items Used

Multicultural acquisition

1. I travel abroad to gain experiences with other cultures.

2. It is important to recognize differences among various cultural groups.

3. I make friends mostly with people of the same cultural origin as mine.

4. I am eager to make friends with people from different cultural backgrounds.

5. Efforts should be made to understand people from different cultural backgrounds.

6. I am curious about traditions of other cultures.

7. I read books or magazines to obtain knowledge about other cultures.

8. I try food from different cultures.

9. One should actively involve himself or herself in a multicultural environment.

10. I learn customs and traditions of other cultures.

11. I am happy to learn the history and geography of other cultures.

Ethnic protection

12. I find living in a multicultural environment very stressful.

13. Speaking another language makes me nervous.

14. I feel isolated from people of other cultural groups.

15. The ways that people of different cultural origins think and act often make me confused.

16. I am worried that people from other cultures would not understand my ways of doing things.

3. Identification with All Humanity (IWAH) items used

(all items ask about “People in my community”, “People in my country” and “People all over the world” in that order for each question stem)

IWAH Bond

1. How close do you feel to each of the following groups?

2. How often do you use the word “we” to refer to the following groups of people?

3. How much would you say you have in common with the following groups?

IWAH Concern

4. How much do you believe in being loyal to…

5. How much would you say you care (feel upset, want to help) when bad things happen to…

6. When they are in need, how much do you want to help:

7. How much do you want to be a responsible citizen of…
